# Edge IoT Prototyping Using Model-Driven Representations: A Use Case for Smart Agriculture

**DOI:** 10.3390/s24020495

**Published:** 2024-01-12

**Authors:** Ivan Guevara, Stephen Ryan, Amandeep Singh, Colm Brandon, Tiziana Margaria

**Affiliations:** 1 Department of Computer Science and Information Systems, University of Limerick, V94 T9PX Limerick, Ireland; stephen.e.ryan@ul.ie (S.R.); amandeep.singh@ul.ie (A.S.); colm.brandon@ul.ie (C.B.); tiziana.margaria@ul.ie (T.M.); 2 Lero, V94 NYD3 Limerick, Ireland

**Keywords:** Industry 4.0, model-driven development, analytics, smart agriculture

## Abstract

Industry 4.0 is positioned at the junction of different disciplines, aiming to re-engineer processes and improve effectiveness and efficiency. It is taking over many industries whose traditional practices are being disrupted by advances in technology and inter-connectivity. In this context, enhanced agriculture systems incorporate new components that are capable of generating better decision making (humidity/temperature/soil sensors, drones for plague detection, smart irrigation, etc.) and also include novel processes for crop control (reproducible environmental conditions, proven strategies for water stress, etc.). At the same time, advances in model-driven development (MDD) simplify software development by introducing domain-specific abstractions of the code that makes application development feasible for domain experts who cannot code. XMDD (eXtreme MDD) makes this way to assemble software even more user-friendly and enables application domain experts who are not programmers to create complex solutions in a more straightforward way. Key to this approach is the introduction of high-level representations of domain-specific functionalities (called SIBs, service-independent building blocks) that encapsulate the programming code and their organisation in reusable libraries, and they are made available in the application development environment. This way, new domain-specific abstractions of the code become easily comprehensible and composable by domain experts. In this paper, we apply these concepts to a smart agriculture solution, producing a proof of concept for the new methodology in this application domain to be used as a portable demonstrator for MDD in IoT and agriculture in the Confirm Research Centre for Smart Manufacturing. Together with model-driven development tools, we leverage here the capabilities of the Nordic Thingy:53 as a multi-protocol IoT prototyping platform. It is an advanced sensing device that handles the data collection and distribution for decision making in the context of the agricultural system and supports edge computing. We demonstrate the importance of high-level abstraction when adopting a complex software development cycle within a multilayered heterogeneous IT ecosystem.

## 1. Introduction

Smart technologies are transforming the agricultural sector by offering innovative solutions to increase productivity, efficiency, transparency, and sustainability. However, incorporating them into the agricultural industry also poses new challenges that need to be addressed. These challenges include the compatibility of new and old platforms, the protection of sensitive electronics from harsh environmental conditions, the regulation and ethics of data collection and sharing, and the inclusion and empowerment of small-scale producers. In parallel with these growing needs, emergent smart agriculture is applying cutting-edge technologies to improve the current agricultural practices regarding better use of the available resources and better information and support along the entire lifecycle, from the soil to the consumer. Techniques such as machine learning (ML), big data analytics, advances in sensors, communications, new machines like UAVs (unmanned aerial vehicles), and advanced software development techniques pave the way to a new agricultural era, where these technologies have the opportunity to boost smart farming to a new level. Several use cases in the field span the use of UAVs for weed detection [1], robotic harvesting spotting pests using IoT [2], or a data forwarding algorithm for monitoring nutrient deficiency [3]. These use cases exploit the opportunities offered by applying one or more of the new technologies to the agricultural production pipeline. From a data analytics point of view, ref. [4] distinguishes several categories of data analytics:

- Memory-level analytics: analysing the data stored in some storage memory statically.
- Massive analytics: also referred to as big data analytics, it is the procedure of analysing large amounts of data in order to extract information.
- Business intelligent data analytics: it uses data to uncover insights that help a company or an organisation. The focus is on both present and future data.
- Offline analytics: it concerns analysing data for decision making but not necessarily on the arrival of the data, in order to derive insights and strategies.
- Real-time analytics: It involves reasoning and statistics on fresh actual data in order to provide faster and immediate rational and well-informed decisions, such as when reacting to sudden events.

The flexible approach to where to store and where to compute that we showcase in this paper allows users to exploit different options in a flexible and low-code/no-code fashion.

### 1.1. Background and Related Work

There are several IoT-based smart systems use cases for agriculture production and to improve food production, such as Bu and Wang [5], which uses a four-layered structure consisting of (1) an agricultural data collection layer, (2) an edge computing layer, (3) an agricultural data transmission layer, and (4) a cloud computing layer. In this context, the information system consists of an *agriculture data collection layer* composed of several IoT devices retrieving different types of data (humidity, temperature, amount of light, etc.), enabling more complete information for better decision making. The next component in the pipeline is the *edge computing layer*, responsible for the data preprocessing from the devices, combining and optimising the different data structures retrieved, and avoiding unnecessary duplication. Once the data are optimised, they are delivered to the *agriculture data transmission layer* to be transmitted to the cloud computing layer and the real-time decision making process through deep reinforcement learning (DRL) models starts, by measuring and calculating the needs for the entire harvest.

The system described in Rubanga et al. [6] delivers a low-cost solution for a tomato greenhouse using wireless sensor network (WSN) devices for micro-climate data and a PC for daily activity data (temperature sensor, humidity sensor, a barometer, a CO_2_ m, and a sound meter). The PC contains a Google Drive spreadsheet where different data are written manually, such as harvest, fertiliser use, and daily activities. Everything is then transmitted to a rental SaaS server using a MySQL database for storage, as well as the sensor data coming from the WSN devices, crop calendar, yield, etc. To analyse the data obtained, the growing degree day (GDD) method is used as a heuristic tool to measure heat accumulation to predict plant and animal development rates [7].

The use case presented by Saleheen etal. [8] consists of a NodeMCU ESP82266 microcontroller acting as the main actuator with a soil moisture sensor (YL-38), a waterproof temperature sensor (DS18B20), a temperature and humidity sensor (DHT11), a barometric pressure sensor (BMP180), a gas sensor (MQ135) for measuring air quality, an LDR light sensor to measure whether the plants are receiving enough light or not, an SIM800L GSM module to send messages to a phone to start or stop irrigation, an LCD 16 x 2 display to indicate output results of the sensors, and finally a two-channel 5 V relay module to turn on/off the water pump and the light source. In terms of software tools, it uses the Adafruit IO IoT-based cloud to interact with the hardware components via the internet and the Arduino IDE to upload the code to the NodeMCU board. The process works as follows: the board collects the data from all the sensors, and then the data are sent to the LCD and Adafruit IO platform. If the soil moisture is less than 15%, the GSM module will send an SMS to the user’s phone to start the water pump; if it is greater than 80%, it will send an SMS to the user to stop the pump. The decision still relies on the user, whether to start/stop the water pump and to turn on/off the light, but the entire pipeline simplifies the process.

On the other hand, Mishra et al. [9] use only three layers to develop an intelligent agriculture system: a sensing layer, a fog computing layer, and a cloud computing layer. The first layer is composed of sensors and actuators deployed across the crop field to systematically sense the physical parameters, such as air temperature, air humidity, solar radiation, soil temperature, and moisture. The second layer comprises one or more servers to provide administrative control of the entire IoT infrastructure of the agricultural field, also addressing the limitations of intermittent connectivity, high latency, and high network bandwidth consumption of cloud-based infrastructure. The nodes collect data from the sensors deployed in the field and perform data cleaning, filtering, aggregation, and fusion tasks. The third layer will consist of several end nodes that communicate the parameters from the field with the fog layer using LoRa communication or receiving actions/commands for actuation from those nodes.

Said Mohamed et al. [10]’s exploration of Industry 4.0 and its application in smart agriculture holds significant potential for real agricultural farms, particularly in the domains of sensors, silage, tillage, and fertiliser management. By incorporating advanced technologies such as humidity, temperature, and soil sensors, as well as drones for pest detection and smart irrigation systems, the agricultural processes can be enhanced for improved decision making [10]. Their proof of concept in smart agriculture uses the Nordic Thingy:53 as a multi-protocol IoT prototyping platform, demonstrating the practical application of these concepts. The integration of advanced sensing devices and edge computing capabilities contributes to efficient data collection and distribution for informed decision making in agricultural systems. This approach not only streamlines software development but also empowers farmers with tools to navigate the complexities of a multilayered heterogeneous IT ecosystem, ultimately improving the effectiveness and efficiency of agricultural processes related to silage, tillage, and fertiliser management.

### 1.2. Introducing a Composable Low-Code Architecture for Agriculture

The previously discussed setups are complex and heterogeneous; i.e., they involve the integration of several technological layers working simultaneously to achieve efficient and cost-effective delivery of the different tasks and also require including various devices (actuators and nodes) with their different transfer protocols, dealing with orchestration challenges when trying to define the behaviour of the system. In this context, our approach simplifies the IT and integration tasks by composing *models of behaviour* to achieve the same results. By introducing the different domain-specific languages (DSLs) as a high-level abstraction for each behaviour and plugging them in, we enable a development cycle where stakeholders, instead of only defining requirements, can take action by participating actively in the successive stages of the project.

In this context, our contributions cover the demonstration of various aspects of the use of two low-code/no-code (LC/NC) development environments, DIME [11] and Pyrus [12], and the extension of their application-domain-specific languages (A-DSLs) to this specific smart agriculture setting. Specifically, we aim to develop a simple, cost-effective smart agricultural system to have some analytics information from a greenhouse and enable a decision making process in terms of the different measures taken from it. To accomplish this, we will extend the range of A-DSLs that these two environments provide, in order to enable our external IoT systems to become amenable to this LC/NC application development approach, allowing a high level of reusability. This extension involves creating several building blocks to communicate with the different actuators, as well as to adopt and integrate the low-cost compact multi-sensor platform Nordic *Thingy:53* [13] that we use to measure the different environmental metrics and enable the analytics pipeline for the subsequent decision making. While this is the component of choice for this demonstrator, the illustrated technology for the integration of IoT sensing hardware and rapid decision making is extensible to other devices. A number of industry partners in our projects are in fact developers of specialised sensors and actuators, and they are keen to lower the adoption threshold of their devices specifically regarding software integration and subsequent application development, which are issues for a large portion of their customer base.

In the remainder of the paper, Section 2 briefly introduces our smart agriculture application, as well as all the components of the tools we use to design and implement it. Section 3 describes the experimental setup, the new methodology based on model-driven design and development, and the software architecture with the corresponding pipelines. Section 4 includes the results and discusses limitations, the flexibility aspects, and the enhanced reusability arising from our choice of specific MDD approach, and, finally, Section 5 contains our conclusions.

## 2. Materials and Methods

In this section, we introduce the case study, the components of our smart agriculture application, and the tools we use to design and implement it.

### 2.1. The Agricultural Setup: IoT and the Edge

The agricultural setup we consider consists of a greenhouse equipped with the Nordic Thingy:53 sensors, which helps us track light, temperature, pressure, humidity, and air quality. While we primarily consider a greenhouse, the same technology could be mounted on movable agricultural equipment, like a tractor, that may be driven by a human or autonomously, or a different controlled environment, like one or more silos where harvested products must be stocked under the proper controlled conditions for that product. Nordic uses the term IIoT as “Industrial IoT” because the sensors are mostly used in the context of industrial processes, like manufacturing floors and warehouses. Here, we welcome the robustness of their sensors for the greenhouse, outdoor, or controlled environments.

While the systems from the literature explicitly introduce heterogeneous layers in the software and systems architecture, we focus instead on a **plug-and-play approach based on the DIME and Pyrus flavours of LC/NC development**, which are more easily extensible and more straightforward to design, implement, and maintain.

The specific subsystems and technologies we consider and introduce are, in order, the IoT platform and device of choice (Section 2.2), the data storage and management in a NoSQL database (Section 2.3), and the choice to adopt LC/NC and MDD technology using two such design environments (Section 2.4).

### 2.2. IoT platforms: Thingy Series

**Figure 1:**
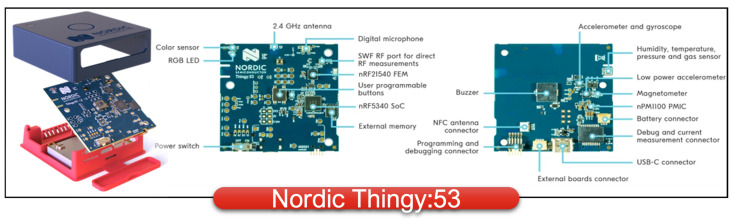
The Thingy:53 sensors and I/O overview.

The Nordic Thingy:53 is an IoT device that allows users to create prototypes of IoT-enabled systems and applications comfortably, without the need to create custom hardware. As shown in Figure 1, Thingy:53 contains a dual Arm Cortex-M33 processor on a *System-on-Chip*(SoC) nRF5340. The processing power and memory size enable it to run embedded ML models directly on the device. It also contains a multi-protocol radio with support for Bluetooth LE, Bluetooth mesh, Thread, and Zigbee. The sensor platform consists of many different sensors, like colour and light sensors, accelerometers, and a magnetometer, all accessible without any additional hardware. The device also has autonomous life by using a USB-C rechargeable 1350 mAh Li-Po battery.

The Thingy:53 is also accompanied by a sister app, Edge Impulse (nRF Edge Impulse: https://play.google.com/store/apps/details?id=no.nordicsemi.android.nrfei (accessed on 3 October 2023)), and a website (Edge Impulse: https://edgeimpulse.com/ (accessed on 3 October 2023)), enabling users to transfer sensor data over Bluetooth LE to a mobile device and upload them to the cloud for training. This is a very appreciated capability as it offers the chance to select an already trained subset of ML models to enable a predictive pipeline and then deploy the models back on the Thingy IoT device for predictions. This capability enables an *edge computing* effect because optimised algorithms can, for example, filter, de-noise, and preprocess those data directly on Thingy at the edge. It is also possible to carry out full edge computing if some trained and optimised models can fit on the Thingy platform and, for example, categorise or classify the sensor data at the point of origin so that the data are processed in situ and never transferred elsewhere.

**Figure 2:**
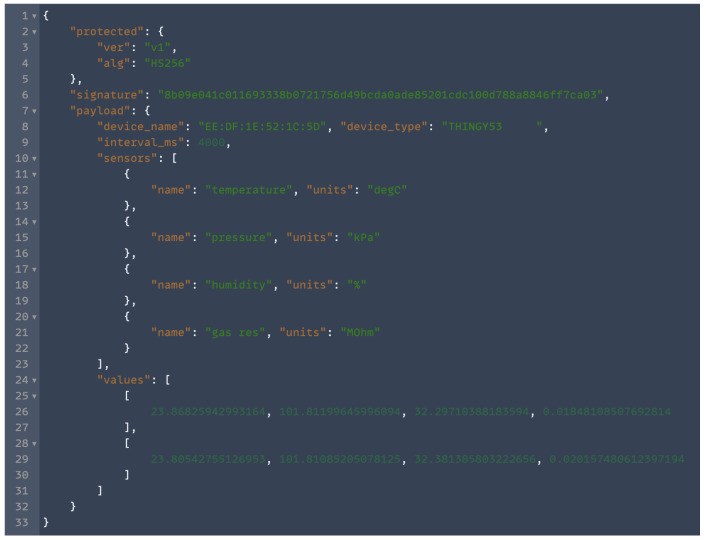
The Thingy:53 sample data description.

The different sensors can be selected for data collection from the smartphone app. The data are originally recorded in CBOR (Concise Binary Object Representation) format. CBOR can be easily converted to more usable and popular JSON and CSV data formats. A snapshot of the data sent to the smartphone app from the Thingy:53 device in CBOR format and after conversion to JSON looks like Figure 2.

The data contain the signature of the device used (unique ID), the name of the device (unique name), the device type (Thingy device), the time interval (in milliseconds) used for data collection, the list of sensors used with units in which values are recorded, and the list of values for each sensor. The values are recorded after a specified time interval, so the time series for the data can be reconstructed by noting the start time of the observations on the smartphone app and appending the time interval for the number of observations.

In comparison with other IoT platforms, such as the Nordic Thingy:52 series, Thingy:53 has twice the processing power and also valuable features like *ARM trustzone*, secure key storage, Bluetooth LE audio, and direction finding. In our use case, we will use the Thingy:53 series, which will allow us to run our ML models efficiently, taking less time for training, testing, and predicting.

### 2.3. Data Storage: No-SQL DB in the Cloud

IoT architectures are inherently intensive in terms of data management as sensors continuously provide a flow of incoming data from multiple sources, creating a large queue of requests to the main application. These data are heterogeneous: they have different types, from basic formats such as images, videos, and text to highly structured types such as JSON formats or entire data structures and complex records. While relational databases can easily handle a huge volume of structured data, they lack enough flexibility to deal with different types of formats in a straightforward way, leading to more complex management, and scaling up, causing very high costs when either is needed [14]. Heterogeneous IoT data need to be stored and managed in such a way that it is possible, and even easy, to extract useful information. Requirements for data management in IoT applications that we need to consider include data heterogeneity, scalability, real-time processing, and security.

On the other hand, as established by [15], MongoDB is an open-source distributed database created in 2007 by DoubleClick (now owned by Google). MongoDB created its own BSON format (binary JSON) and stores the data using a key–value pair strategy, with efficient support for query and retrieval of heterogeneous and even unstructured data in a fashion similar to the object-oriented paradigm. Data can be replicated using the master–slave model, and it also has its own query language that implements all CRUD (create, read, update, delete) operations. As a consequence, we opted to use it in our proof of concept to leverage its main features, and it is the option we feature in the adopted system architecture.

### 2.4. Model-Driven Technologies

We embrace a LC/NC software development paradigm [16], that is rapidly gaining foot in industry and is predicted to become the development style of choice for 80% of newly developed software by 2026 [17]. However, we specifically adopt a Model Driven Design and development paradigm [18,19] where the models are not just graphically suggestive but also have an underlying formal model in terms of Kripke Transition Systems [20]. This choice makes them analyzable through meanwhile well-established techniques like control flow and data flow analysis, model checking [21], property checking, reachability analysis and more, like synthesis [22], also in robotics and IoT contexts [23]. Specifically, we use here the two Integrated Modelling Environments DIME and Pyrus.

**DIME** is an Eclipse-based LC/NC graphical modelling environment that enables prototyping web applications in a model-driven way. It follows the OTA (One Thing Approach) [24] and the XMDD (eXtreme Model-Driven Design) [18] paradigms for modelling and development, empowering domain experts to model an end-to-end web application with no programming experience. To cover the different aspects of web applications, DIME provides a collection of ready-to-use modelling languages, as well as collections of native domain-specific languages (DSLs) that support the development of new applications via composing models of different natures. In particular, DIME allows users to define the data model, create the user interface models, and create the workflows. Those models are checked for syntactic compliance largely automatically (DIME can be downloaded from https://scce.gitlab.io/dime/ (accessed on 3 October 2023)).

**Pyrus** is a web application that offers a graphical, collaborative development environment for data analytics. It bridges the gap between Python-based established programming platforms like Jupyter [25] and graphical workflow composition in a data-flow fashion. Individual Python functions are implemented and stored in Jupyter, and special signature annotations are added to these functions and exported to Pyrus so that the functions can be identified and retrieved by the Pyrus web-based orchestration tool, where the pipelines are composed. From the pipelines, Pyrus generates the Python code for the orchestration and configuration, which is again stored and executed in Jupyter. This separation of concerns decouples the coding and development of the single functionalities (in Python/Jupyter), and the low-code part of the approach, from the data analytics orchestration modelling, which happens in accordance with model-driven engineering principles and completely graphically, in a no-code fashion.

## 3. Software Architecture for the MDD SmartAgri Application

In this section, we present in detail the various steps of the LC/NC application design and implementation, covering our main contributions. Specifically,

- Section 3.1 introduces the runtime architecture we adopted for the SmartAgri demonstrator, highlighting in particular the *Digital Thread* it contains.
- Section 3.2 showcases how we encapsulate Python and Java functionalities to *reusable components*: the SIBs, in the *low-code* aspect of the design methodology.
- Section 3.3 and Section 3.4 show how the Digital Thread *workflows* are designed in Pyrus and DIME, respectively. This is the logic layer of the applications, and it is amenable to *flexible reconfiguration* in a *no-code* fashion.
- Section 3.5 discusses the option of extending the current architecture with machine learning on the edge, enabling *edge-IoT*.

### 3.1. The SmartAgri Architecture with Digital Thread

**Figure 3:**
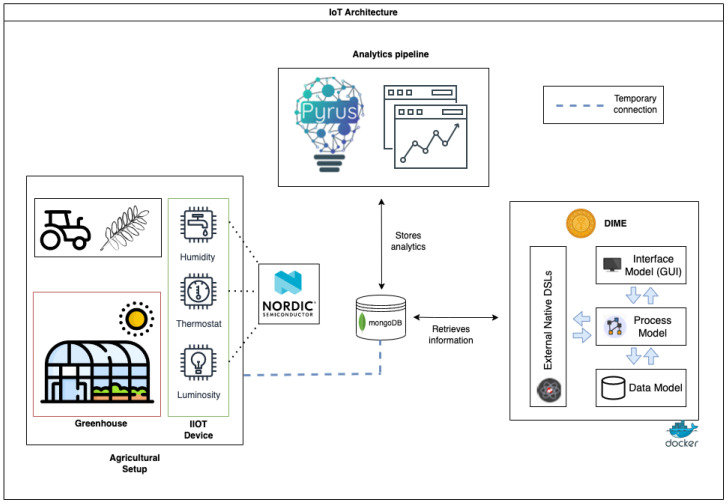
The proposed SmartAgri runtime architecture.

Considering our case study and its components in this context, the system’s architecture for the *SmartAgri* demonstrator is shown in Figure 3. It is composed of three subsystems:

1. the **Agricultural Setup**, consisting of the greenhouse equipped with the Nordic Thingy:53 sensors. The generated data streams from different sensors, such as light, temperature, pressure, humidity, and air quality. The Thingy:53 IoT package is composed of the IoT sensor device itself plus an associated app already developed by the Thingy team, where users can customise the sensor’s behaviour and manage the connectivity through different protocols (Bluetooth LE, WiFi, Zigbee, etc.) in order to gather the generated data. One of the downsides of using Thingy is that, in order to forward the data to another infrastructure, we need to build our custom firmware, so for now we have to upload our collected measurements to the database using a batch script.
2. the **Analytics Pipeline**: Once the data coming from the app are stored in the MongoDB Atlas database, the analytics pipeline designed in Pyrus retrieves them and generates the desired graphics and insights, enabling an informed decision making process and subsequent actuation.
3. the **Digital Thread layer**: a collection of DIME application DSLs define which functionalities the different tools provide. This enables the user to define how they communicate and interact. This happens through a collection of models that implement user interaction and the orchestration of the functionalities of the overall end-to-end system. Specifically, in the Digital Thread, the data are retrieved from MongoDB through the different *REST API external DSLs*, they are analysed through the Pyrus pipeline (in Python), and a dashboard is displayed in a web application defined in DIME, where the stakeholders can visualise the strategic information and make decisions.

To ease understanding, we now provide a detailed description from the bottom up, starting from the components and their integration in DIME through external DSLs.

### 3.2. External DSLs: The Reusable SIBs

The model-driven development environment used in this context, as mentioned before, already incorporates collections of predefined functionalities previously developed and tested. This simplifies the development cycle with different native components, such as those used for interaction with IoT devices (EdgeX [26]), MongoDB, and the integration of external services through REST using the external R services [27]. These DSLs provide convenient high-level abstractions that have a uniform syntax and representation. They help designers of new applications to focus on the “what” instead of the “how” of their own application. This means that developers can focus on the problem, developing the application logic instead of dealing with manual implementation details, and the code of the full application is generated from the models of the logic, data, and UI plus the DSLs, making the entire development lifecycle less prone to errors.

**Figure 4:**
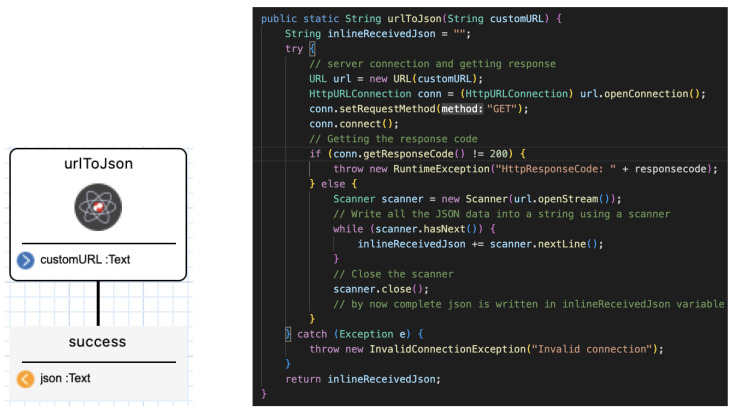
The native common SIBs urlToJson in DIME: SIB representation (**left**) and its corresponding native Java code (**right**).

The DSLs consist of collections of building blocks called SIBs (service-independent building blocks). Figure 4 shows the urlToJson SIB in DIME, which takes a URL and transforms it into JSON format. The SIB appears in the DSL in DIME as a graphical representation of an atomic SIB, with its name and its input/output signature (on the left). It is associated to DIME with a call to its implementation, in this case consisting of plain Java code (JDK 11) (on the right). In the code, the SIB developer has full control over the functionality and the implementation. The representation exposes the I/O and the parameters of the SIB, which makes it easy to reuse in different contexts by changing the parameters. The visual approach to application development makes the system development more user-friendly: developers simply drag and drop the corresponding components into the working area and compose the orchestrations and workflows this way, with full visibility and control of the control flow and the data flow.

### 3.3. The Analytics Pipeline in Pyrus

To design and implement our analytics pipelines, we first use the *Pyrus* LC/NC environment. A DIME version leads to the results presentation shown in the figure in Section 4.4.

**Figure 5:**
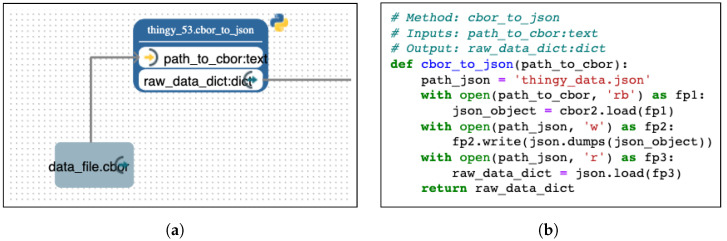
Pyrus: SIB representation of the cbor_to_json() function of the Pyrus pipeline. (**a**) The SIB representation in Pyrus. (**b**) The Python implementation with signature annotations.

Similar to DIME, Pyrus follows an LC/NC approach, but it is a pure data flow tool for analytics applications. As shown in Figure 5 for the cbor_to_json() function of the Pyrus pipeline, underneath each SIB, the implementation is in Python code. On the left, we see the SIB representation in Pyrus, including the SIB name and the input/output signature, and, on the right, we see the Python implementation with the signature annotations for Pyrus. We notice the one-to-one correspondence between the model I/O and the signature annotations augmenting the Python code. The implementation is standard Python.

**Figure 6:**
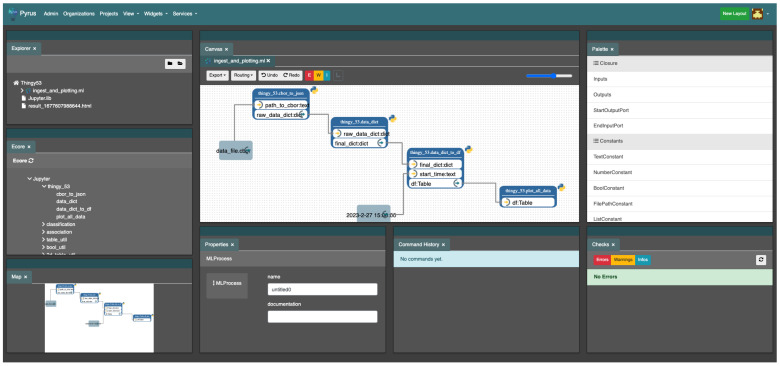
Pyrus: the user view of the web-based development environment.

Figure 6 shows the web-based Pyrus workspace. On the left section, we see it visualise the folder structure of the project. The central canvas shows the workflow of the current pipeline itself, and underneath the canvas its properties, and the right section shows the SIB palette: these are the different SIBs that are ready and available for inclusion in the project.

**Figure 7:**
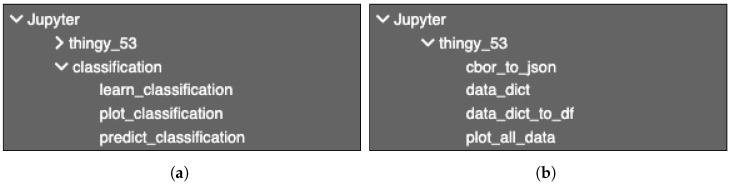
Pyrus: the lists of SIBs in the Ecore palette. (**a**) Sample default SIBs in Pyrus, here the classification palette. (**b**) Custom SIBs used in Pyrus: the new thingy_53 palette.

To implement the pipeline for our smart agriculture case study, some functionalities were missing and thus new SIBs were developed (code and SIB definition), and other existing SIBs needed to be customised in order to deal with new types of extensions, such as CBOR files (Concise Binary Object Representation), that were not supported in Pyrus so far. As shown in Figure 7, the thingy_53 palette is new, and contains among others a new SIB to convert the CBOR data to JSON. It is the same SIB whose representation and implementation are shown in Figure 5.

**Figure 8:**
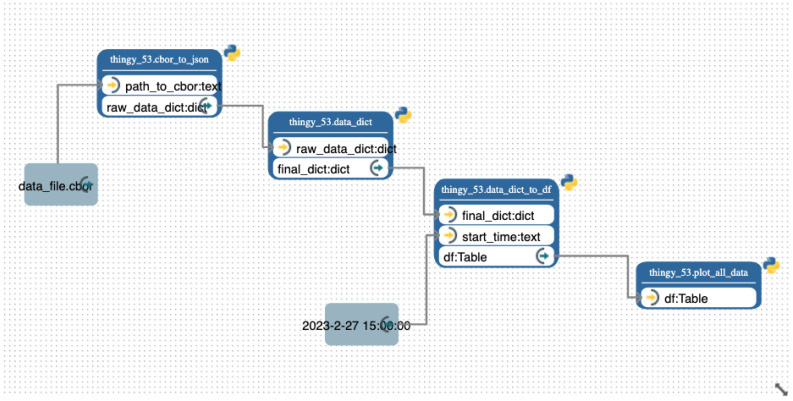
Pyrus: the preprocessing and plotting pipeline.

**Figure 9:**
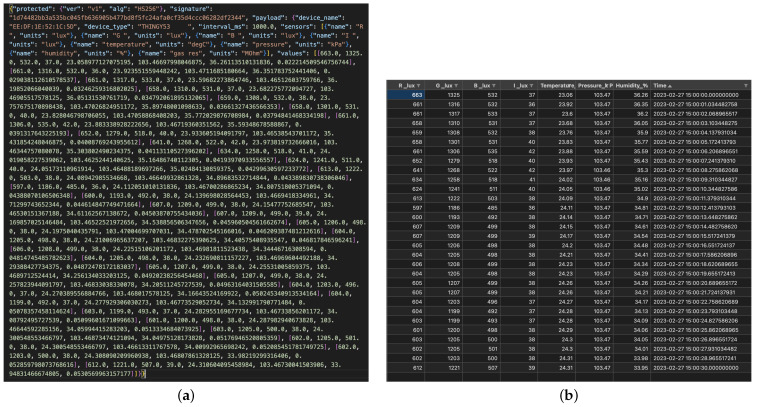
The Thingy:53 datasets before (**a**) and after (**b**) the Pyrus preprocessing and plotting pipeline. (**a**) Test JSON data before processing in Pyrus. (**b**) Test CSV data after processing in Pyrus.

**Figure 10:**
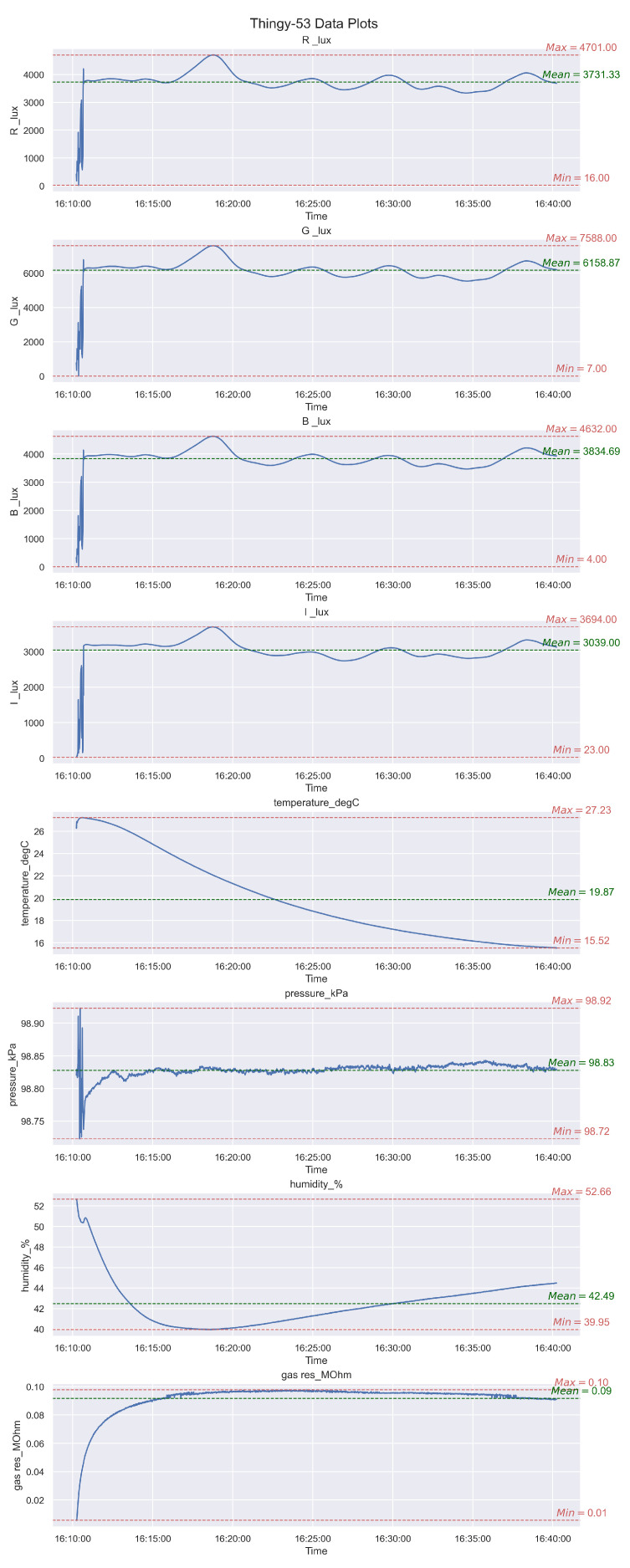
Pyrus: results from the processing and plotting pipeline.

The entire preprocessing pipeline shown in the Pyrus canvas in Figure 6 (middle) is reported for clarity again in Figure 8. It consists of obtaining the input CBOR file, transforming it into JSON format, then creating a dictionary from that file, thus making the transition to a Pandas DataFrame [28] more straightforward, and finally plotting the data. The before/after display of the data structure is shown in Figure 9: on the left is the original JSON, and on the right is the final CSV data format. We can clearly see in this example the benefits of dealing with data structures compared to raw data. This user-friendly approach abstracts through Pyrus SIBs and Pyrus workflows the coding complexity we might find in a regular pure Python approach, and it boosts ease of understanding and reusability, where the system development is based on the easy composition of reusable pipeline components and models. See also Figure 10.

### 3.4. The Analytics Workflow in DIME

**Figure 11:**
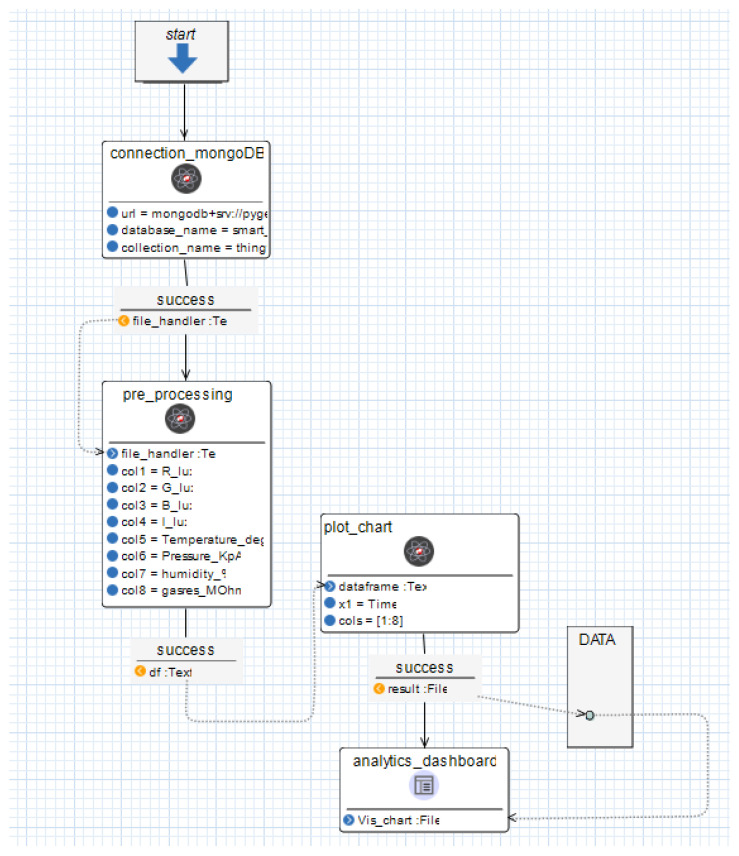
DIME workflow: sample analytics dashboard using R in DIME.

The analytics workflow depicted in Figure 11 serves as a blueprint for a web application for conducting numerical and statistical data analysis. To initiate the workflow, the SIB connection_mongoDB dispatches instructions to the R server, alongside the necessary inputs: the connection string, database name, and collection name of the MongoDB database. The R environment, utilising the mongolite package, retrieves the dataset in JSON format and provides a file handler for the dataframe.

This file handler is then transmitted to the pre_processing SIB, along with the column names requiring segmentation. The R server proceeds to segment the primary dataframe into several sub-dataframes based on the specified inputs. In this instance, the sub-dataframes are R_lux, G_lux, B_lux, L_lux, Temperature_degC, pressure_KpA, humidity_%, and gas_res_MOhm, corresponding to the 8 sensors. These sub-dataframes are subsequently forwarded to the plot_chart SIB, each for a specific sensor.

The generated plots are then shared with the analytics_dashboard’s GUI SIB, which in turn displays them on the web application dashboard.

The solid arrows depict the control flow of the application, the dashed arrows the data flow, analogous to the Pyrus convention.

This is the modelling level where the flexibility of the workflows plays a role: in case one wishes to modify the workflow, e.g., add processing steps, notifications, logging, etc., it is easy to add existing SIBs and “re-wire” the workflow graphically, without the need to master programming skills or a specific (set of) programming languages. This is the no-code aspect of the MDE software development paradigm.

### 3.5. ML on Edge Impulse

**Figure 12:**
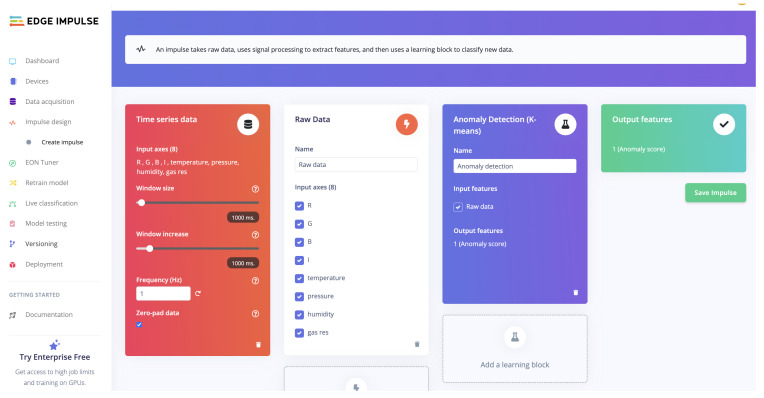
Edge Impulse: LC/NC workflows for data processing, analytics, and ML modelling.

One of the advantages of using the Thingy:53 device and the accompanying platform is the option to send the data to the cloud for data cleaning, feature selection, ML/deep learning (DL) modelling, ML/DL model evaluation, ML/DL model testing, and deploying the selected ML/DL model back on the Thingy device. The Edge Impulse platform shown in Figure 12 is an LC/NC platform for defining workflows. Referred to as ‘impulses’, these workflows connect the *data block* containing the time series collected by the device to the *data processing block*, which then can be linked to the *learning block* for ML/DL model selection. Once the impulse is saved, these blocks have their own full-page options to configure through parameters details such as scaling, filtering, feature selection, as well as modelling parameters, such as epochs, batch size, number and type of layers in the neural network, etc. On the free version that is available to all, Edge Impulse can train a model for a maximum of 20 min training time, which seems a short time, but it allows for many IoT applications in various domains. One such application is the Human Activity Recognition (HAR) domain as discussed by Singh et al. [29], where the authors compared the performance of traditional ML modelling techniques to the DL methods available on Edge Impulse using the data collected using Thingy:53 devices. There, the training time limit of 20 min on Edge Impulse did not limit the options to train a DL model that compares in performance with the traditional ML models trained on local machines. Once more, data are collected for the different sensors utilised in this research, and a successful ML/DL modelling pipeline can be developed and then deployed back on the Thingy:53 device for live predictions on the edge.

## 4. Results and Discussion

The main result is the demonstrator itself, as an application implemented with the new LC/NC technologies, that we can showcase to adopters, research exhibitions, and commercial fairs, and we can even bring it to schools or career days to attract younger students to choose a profession that is related to IoT and analytics.

### 4.1. Modelling a Pipeline

As mentioned previously, Pyrus provides functionality already developed and tested, and a no-code way to simplify the development process: by dragging and dropping the SIBs in our analytics pipelines. An example of this is shown in Figure 7a and Figure 7b, respectively, where we use functionality from the Pyrus core (fit data, plot, and predict) and developed customised functionality as well for our use case: an SIB for converting from CBOR format to JSON, an SIB to create a Python dictionary from data, another one to convert the dictionary into a *Pandas dataframe*, and the last one, a function to plot all our obtained data. The resulting dashboard produced by the Pyrus pipelines is shown in Figure 8, which visualises the pipeline composition with the following stages:

1. Place the data file from the Thingy data_file.cbor into the system. The Thingy devices record data in CBOR format, which is unwieldy for our purpose.
2. We convert the CBOR data file to a more accessible JSON format using the cbor_to_json SIB, and save it in a JSON file.
3. Using the data_dict SIB, the JSON file is initialised as a Python dictionary, and the information that is not needed for data analysis is discarded. For example, the ID numbers for different sensors were discarded in favour of their names.
4. To obtain more precise context for the experiment, we clean the data by converting the time series format to Python *datetime* and initialise the dictionary as a Pandas DataFrame for ease of processing, storage, and plotting. This is achieved in the data_dict_to_df SIB.
5. Finally, we plot all the obtained data as shown in Figure 10.

### 4.2. Dashboard and Analytics

In Figure 10, only a subset of the dataset (corresponding to approximately 30 min) was taken for demonstration of the pipelines.

- The first three graphs titled R_lux, G_lux, and B_lux are from the colour sensor on the Thingy:53. These plots show the different colour values for the red, green, and blue channels, respectively. These values are important to consider, especially in a controlled environment like a greenhouse, because the growth of several plants is strongly related to the colour of the light exposed to them.
- The fourth graph in the figure is from the light sensitivity sensor and is used to measure the amount of light the plants are exposed to.
  The data from these four graphs can be used to find the most efficient wavelength a particular plant responds to or expose the plants to a certain light for a certain amount of time.
- The next three graphs in the figure titled temperature_degC, pressure_kPa, and humidity_% plot the measurements of temperature (in degrees Celsius), pressure (in kilopascal) and humidity (in percentage), respectively, in the greenhouse. These values can be tightly controlled by using heating, air conditioning, humidifiers, etc., to achieve the optimum growth for the plants. As indicated in the graphs, the temperature and humidity values fluctuated quite a bit, which meant that the greenhouse environment was not as tightly controlled as assumed beforehand.
- The last graph in the figure shows the measurements from the gas sensor and is necessary to evaluate the quality of air inside the greenhouse.

### 4.3. Limitations

A few limitations with the Thingy:53 and its supported software stack emerged. For example, the device cannot directly upload to cloud storage, and it also needs the smartphone app to connect/control every basic functionality. To change this behaviour, as already mentioned, custom firmware would have to be written, which was outside the scope of our project. Another small but significant issue with the smartphone app is that the duration of data measurement had to be entered in milliseconds using increments/decrements of one millisecond. So, to set up the data collection for a 30 min duration, it would take around 15 min just to set the correct number in milliseconds. There was no way of directly entering the exact figure using the keyboard. This issue hindered the data collection step the most and could be solved with just a simple UI update from Thingy’s developers.

### 4.4. Flexibility

**Figure 13:**
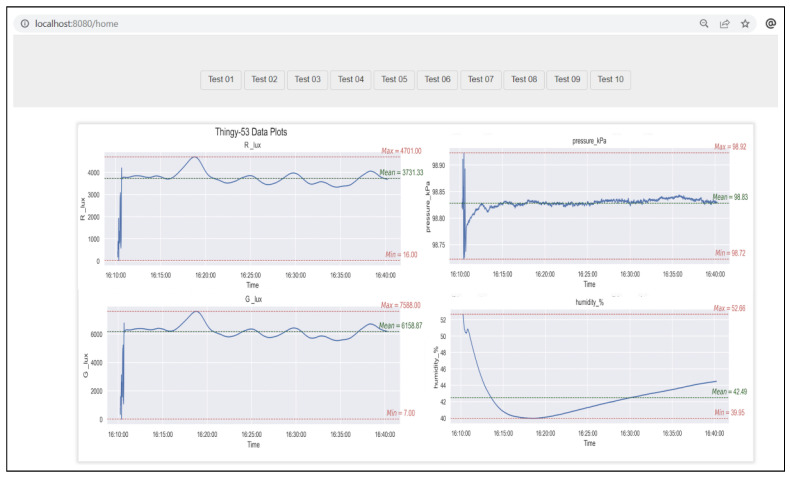
DIME: the smart agriculture sensor data dashboard as shown in the web application.

From the point of view of observing and logging the changes in different parameters in the greenhouse, the current setup, including the Thingy:53, the smartphone app, Pyrus pipelines, and the DIME web app, was very successful. It was possible to observe and analyse the data from the different sensors in great detail. The results dashboard was produced by the Pyrus pipelines and fed into the DIME dashboard (Figure 13), enabling a more user-friendly, web-based approach. There, we chose to display only the red and green light channels, pressure, and humidity values as they were sufficient in order to monitor the environmental parameters in our greenhouse and to make decisions: for example, if the humidifier was to be turned on or not, or if the coloured lights were to be switched on or off, and so on.

With this new methodology for designing, implementing, and deploying applications, once having SIBs that conduct, for example, data cleansing, noise elimination, etc., for which we can reuse existing Python code or Java code and wrap it as shown in Section 2.4, the Digital Thread workflows designed in Pyrus and DIME constitute the logic layer of the applications. This logic layer is amenable to flexible reconfiguration in a no-code fashion to add, for example, alternative sub-workflows, e.g., for data cleansing, noise elimination, etc.

### 4.5. Reusability

The produced SIBs, processes, and features like the Pyrus pipeline also have the chance to be reused:

- in a *no-code fashion* if reusable is possible as is, i.e., no new coding is needed. This is the case if one applies the same Pyrus pipeline and DIME application to produce different plots if the input data are different. However, it is also the case if there is a redesign of either the Pyrus analytics pipeline or the DIME data collection, analytics orchestration, and dashboard application in case we have all the SIBs we need. Now that we have extended the DIME and Python SIBs to deal with several Nordic Thingy:53 data types and functionalities, we could create very different pipelines for different applications without the need of being able to program or master the software development lifecycle through subsystem integration and application deployment and provisioning. For example, if instead of providing the entire data plot of the luminosity or humidity we wanted only to expose the times above/below given thresholds (which may be needed to automate the decision making, and also easier to understand by a garden or greenhouse operator), we would modify the current Pyrus workflow by reusing already existing SIBs that handle data filtering regarding thresholds and display.
- in a *LC fashion*, if we wished, e.g., to extend the collection of Nordic Thingy:53 functionalities integrated in DIME further, extending the corresponding DIME and/or Pyrus DSL in a fashion similar to what we completed so far. Here, we count on the powerful help of the examples of the current DSL in order to simplify this task: it is easier to create a new SIB on the basis of open-source SIB collections made available to the community of DIME and Pyrus users than to have to create it from scratch as we did.

The reusability aspect, alongside the no-code feature of the development routine, makes it accessible for end-users who are not proficient in the use of programming languages. This trait not only helps speed up the development cycle for a simple data collection and analysis project for a small greenhouse but also democratizes the use of IoT sensors for all. Previous work has already been done in several domains utilising precursor technologies where we can witness the effectiveness of this approach. In the context of bioinformatics we can find for instance [30], in the Telecommunications domain [31], Robotics and IoT [23], and Decision support systems [32].

## 5. Conclusions

In this case study, we developed and deployed an edge analytics solution using a multi-sensor IoT platform that takes the data from greenhouse stores and analyses the different data streams we need. Through a Python script, we downloaded the data and stored them in a no-SQL database (MongoDB) in order for the Pyrus data analytics to create the individual sensor data graphics and provide them to the web application as elements to display in a dashboard for decision making, in our case for the greenhouse operation optimisation. We used DIME to orchestrate the overall web application, in particular to show the results, leveraging the already built-in DSLs and SIBs in our application design and implementation. Our innovative platforms and this development approach have the potential to greatly simplify the development and deployment of such applications.

All the setups from the literature mentioned in Section 1.1 are complex and heterogeneous: they involve the integration of several technological layers working simultaneously to achieve efficient and cost-effective delivery of the different tasks and also require including various devices (actuators and nodes) with their different transfer protocols, dealing with orchestration challenges when trying to define the behaviour of the system. In this context, our approach simplifies the IT and integration tasks by composing *models of behaviour* to achieve the same results. By introducing different domain-specific languages (DSLs) as high-level abstraction for each behaviour and plugging them in, we enable a development cycle where stakeholders, instead of only defining requirements, can take action by participating actively in the successive stages of the project.

LC application development environments make a great contribution to the underlying model-driven development and generative paradigms. They do so by facilitating the bootstrapping and composability of individual functional components, and, in this way, they make LC/NC an increasingly popular approach to planning, designing, and developing non-conventional heterogeneous Digital Thread ecosystems that include sensors and decision making based on more or less sophisticated analytics. The growing diversity in technical expertise needed in order to deliver high-tech solutions and processes in a conventional way, by direct manual coding, renders the idea of using these no-code alternatives increasingly attractive. Not only does it empower domain experts with no programming capabilities and make them full participants in the development process but it also reduces costs in terms of development speed, testing, deployment, and adoption.

The next steps include, on the adoption and demonstration side, the uptake of the case study within the Confirm Irish Research Centre on Smart Advanced Manufacturing. This case study is in fact a practical demonstrator of the Digital Thread platform arising in the Confirm Research Hub on Cyberphysical Systems. Due to the strong importance of advanced agriculture in the Irish economy, with its global export of premium products, we have confirmation from many industry partners from the smart agriculture sector who are interested in these new software development technologies. Also, the new governmental initiatives towards mainstreaming organic farming, spanning both produce and livestock, have raised the interest and relevance of advanced real-time ambient and conditions monitoring for indoor and outdoor environments. Applications are, e.g., to reduce water and energy consumption as well as to reduce or eliminate the preventive use of pesticides and fertilisers.

On the technical side, we intend to extend and refine the overall architecture for this application, in order to better enable the integration between the physical world (IoT multi-sensor device) and extend the analytics pipeline and the Pyrus/DIME dashboards. The largest challenge ahead is likely the firmware-level development needed to configure the Thingy in a more efficient way, and, finally, we intend to fine-tune the dashboard in order to enable better and smoother interaction with users.

## Data Availability

The data are located publicly in the following repository: https://doi.org/10.5281/zenodo.7712904) (accessed on 3 October 2023).
